# Knowledge, Attitude, Practices, and Vaccine Hesitancy Among the Latinx Community in Southern California Early in the COVID-19 Pandemic: Cross-sectional Survey

**DOI:** 10.2196/38351

**Published:** 2022-08-04

**Authors:** Shivani N Mehta, Zoe C Burger, Stephanie A Meyers-Pantele, Richard S Garfein, Dayanna O Ortiz, Pavan K Mudhar, Smit B Kothari, Jigna Kothari, Meena Meka, Timothy Rodwell

**Affiliations:** 1 San Diego School Of Medicine University of California La Jolla, CA United States; 2 Amistad Medical Clinic Santa Ana, CA United States; 3 Department of Psychology San Diego State University San Diego, CA United States; 4 Herbert Wertheim School of Public Health University of California, San Diego San Diego, CA United States; 5 Department of Audiology Arizona School of Health Sciences Mesa, AZ United States; 6 University of Rochester Rochester, NY United States

**Keywords:** COVID-19, knowledge, attitude, practices, KAP survey, vaccine hesitancy, Latinx, Latinx cohort, minority population, primary care, sociodemographic characteristic, public health, vulnerable population, epidemiology

## Abstract

**Background:**

The Latinx population in the United States has experienced high rates of infection, hospitalization, and death since the beginning of the COVID-19 pandemic. There is little data on the knowledge, attitude, and practices (KAP) specifically in Latinx communities in the United States.

**Objective:**

We aimed to assess COVID-19 KAP and vaccine hesitancy among a Latinx cohort in the early stages of the COVID-19 pandemic (from July 2020 to October 2020), at a unique time when a vaccine was not available.

**Methods:**

Participants aged ≥18 years were recruited at a primary care clinic in Southern California and asked to self-report sociodemographic characteristics, KAP, and vaccine hesitancy. A subset of the participants answered the vaccine hesitancy assessment as it was added after the start of data collection. KAP items were summed to create composite scores, with higher scores reflecting increased COVID-19 knowledge, positive attitudes toward the COVID-19 pandemic, and disease prevention practices. Bivariate and multivariable regression models were fitted to test associations between sociodemographic characteristics and KAP scores. For our analysis, we only included patients who self-identified as Latinx.

**Results:**

Our final data set included 265 participants. The participants had a mean age of 49 (IQR 38.5-59) years, and 72.1% (n=191) were female, 77% (n=204) had at most a high school degree, 34.7% (n=92) had an annual income <US $25,000, and 11.7% (n=31) had previously tested positive for COVID-19. We found high knowledge regarding transmission and spread; moderate knowledge regarding symptoms awareness; overall negative attitudes, which included high pessimism in government public health efforts and high amounts of fear, anxiety, and frustration due to COVID-19 pandemic; and moderate participation in preventive practices. A college education was positively associated with a higher knowledge score than those without a college education (β=0.14, 95% CI 0.01-1.60; *P*=.04) when adjusted for covariates. Male gender had a positive association with COVID-19 attitude scores compared to female gender (β=1.61, 95% CI 0.50-2.72; *P*=.05), and male gender was negatively associated with the COVID-19 practices score compared to female gender (β=–0.16, 95% CI –0.56 to –0.06; *P*=.03), when both were adjusted for covariates. Among a subset of 203 patients, 26.6% (n=54) indicated that if the vaccine was available, they would not take a COVID-19 vaccine, and 18.7% (n=38) were unsure.

**Conclusions:**

Good knowledge and preventative practices in the population may have reflected effective public health messaging and the implementation of public health laws during the first wave of the pandemic; however, the overall fear and anxiety may have reflected the negative impact that the pandemic had on vulnerable populations such as the Latinx community. Although our data are a reflection of a previous time in the pandemic, we believe it captures a critical time that can be used to provide unique insights regarding potential avenues to better protect the Latinx communities against future vaccine-resistant COVID-19 strains.

**International Registered Report Identifier (IRRID):**

RR2-10.2196/25265

## Introduction

In early 2020, the global community was overwhelmed by the COVID-19 pandemic caused by the SARS-CoV-2 virus, leading to quickly rising incidence and death rates with no available vaccine. By December 2020, COVID-19 had infected almost 20 million people and caused more than 300,000 deaths in the United States [1]. As cases of COVID-19 climbed across the United States throughout 2020, racial/ethnic minority populations were more likely to be infected, experience complications, and die from COVID-19 than the general population during a time when the vaccine was not available [2]. Although Latinx populations made up only 38.9% of the total population in California, they accounted for almost 60% of COVID-19 cases and approximately 50% of COVID-19–related deaths during the early pandemic in 2020 [3].

In 2021, the Moderna and Pfizer vaccines were made accessible to thousands of Latinx individuals in California [4], especially due to outreach and advocacy by organizations such as the Latin Community Foundation, Hispanic Federation, and the Orange County Public Health Department [5-7]. Despite this important milestone in the pandemic, the Latinx, Black, and American Indian or Alaska Native communities had similarly slow rates of vaccine uptake throughout 2020 and 2021 compared to Asian and White populations. As expected, Black and Latinx communities reported increased vaccine hesitancy compared to White and Asian populations in the United States [8,9].

The Latinx community remained one of the most vulnerable groups in terms of infection and death rates compared to the rest of the state population in the emergence of the new Omicron variant [10].

As we enter the third year of the COVID-19 pandemic, we still have not fully investigated the risk factors that predisposed the Latinx population in California to significantly higher incidence rates in the first year of the pandemic compared to other communities. In addition to the greater lack of access to care in the Latinx community, there are many other structural disparities that contribute to decreased health equity and increased risk behaviors in this population [11-14]. The Knowledge, Attitude, and Practices (KAP) theory is a health behavior theory based on the belief that knowledge is the foundation of behavior change and helps form attitudes and practices that drive behavior change [15,16]. Numerous global studies from the beginning of the pandemic showed that a lack of COVID-19 knowledge, negative attitudes toward the pandemic, and the lack of disease preventative practice were more common in communities with high disease rates [14,17-21]. Further, vaccine hesitancy has been a persisting issue for the last few decades during the presence of numerous pandemics, especially within minority populations [22,23]. In the beginning of the COVID-19 pandemic, few studies measured the vaccine hesitancy of a potential COVID-19 vaccine before the vaccine was made available [24,25]. It is possible that if this data was measured early on, there would be more information to assist public health programs to combat vaccine misinformation and mistrust at the start of the vaccine release to the public [26]. There have been a small number of other studies that have explored the differences in KAP between different ethnic groups and genders in the United States, but none have specifically focused on the Latinx population.

In this study, we sought to investigate COVID-19 KAP and vaccine hesitancy among a Latinx population in Southern California. Specifically, we aimed to capture information on KAP and vaccine hesitancy from a critical time early in the COVID-19 pandemic when the vaccine was not available. The findings of this work provide unique insights regarding vulnerabilities and potential avenues for better protecting Latinx communities in future pandemics and against new vaccine-resistant COVID-19 strains, such as the currently circulating Omicron strain.

## Methods

### Study Design

We conducted a cross-sectional study from July to October 2020 in Southern California, during the “stay at home” order period that was implemented by the state government of California. We carried out our surveys at 2 primary health care centers in Santa Ana and Anaheim, California. Using consecutive sampling methods, we predominately recruited and surveyed patients presenting for telehealth appointments but also recruited patients presenting for in-person appointments at the clinics. Patients were eligible for study participation if they were (1) aged ≥18 years and (2) an enrolled patient of Amistad Medical Clinic Santa Ana or Amistad Medical Clinic Anaheim. For this analysis, we only included participants who self-identified as Latinx. Our study design, which included in-person and telemedical recruitment, and data collection methods have been previously described [27].

### Survey Validation

Our KAP survey was adapted from the World Health Organization’s (WHO) Guide to Developing Knowledge, Attitude, and Practice Surveys [15], in addition to the few existing global COVID-19 KAP studies from the time period [28-30]. The most current COVID-19 symptoms included in the knowledge survey were identified using Centers for Disease Control and Prevention symptom reports that were the most current at the time of data collection. No standardized questionnaire for COVID-19 KAP had been created at the time of the study due to the early nature of the pandemic. Therefore, we pretested our KAP survey among 10 participants before implementing it.

Questions about vaccine hesitancy were added to the survey after the start of data collection. The vaccine hesitancy questions were adapted from the Vaccine Hesitancy Scale by WHO’s Strategic Advisory Group of Experts on Immunization [31]. The survey was pretested among 10 participants before including it in our data collection.

### Measures

#### Sociodemographic Characteristics

We asked participants to self-report sociodemographic characteristics including age (in years); gender (male vs female); education level completed (elementary school, middle school, high school, college degree, or graduate degree); annual income (less than US $25,000, US $25,000 to US $49,999, US $50,000 to US $99,999, and US $100,000 or more); marital status (single/never married, married, divorced, separated, or widowed); ethnicity (Latinx vs non-Latinx); and employment status (currently employed, recently unemployed or on temporary leave due to COVID-19, unemployed before COVID-19, retired, or a student). We also asked participants to self-report whether they had ever tested positive for COVID-19 (yes vs no).

#### COVID-19 KAP Survey

The COVID-19 KAP survey was comprised of 3 parts: (1) COVID-19 knowledge, (2) COVID-19 attitudes, and (3) COVID-19 practices. Knowledge included questions about disease transmission and spread through 12 dichotomous (yes vs no) questions and 1 open-ended question. It also measured knowledge of the most common COVID-19 symptoms through a multiple mark–style question in which participants were able to endorse 1 or more symptoms. All items were then summed to create a composite knowledge score ranging from 0 to 12, with higher scores indicating greater COVID-19 knowledge. Correct answers were based on current COVID-19 data at the time that was disseminated by the WHO to the general public [32].

COVID-19 attitudes were assessed through 5 items regarding confidence in the government control of the pandemic and emotions about the pandemic. All items were measured on a 5-point Likert-type scale ranging from 1 (strongly disagree) to 5 (strongly agree) and were summed to create a composite attitude score, ranging from 5 to 25, with lower scores indicating more negative attitudes. COVID-19 practices consisted of 2 items that assessed participants’ engagement in behaviors that reduce COVID-19 transmission. Both items were measured using a 5-point Likert-type scale ranging from 1 (never) to 5 (always) and were summed to create a composite practices score, ranging from 2 to 10, with higher scores indicating greater engagement in COVID-19 preventative behaviors.

#### COVID-19 Vaccine Hesitancy

Additionally, a question regarding vaccine hesitancy was added to the study after the start of data collection, and a subset of participants provided information on their willingness to take a potential COVID-19 vaccine through their responses (yes, maybe, or no) to the following question, “If a COVID-19 vaccine was available, would you get one?” Those who indicated “no” or “maybe” to this question were then asked to report their reason for their vaccine hesitancy. Response options included “I don’t think it would work,” “I’m concerned about the possible side effects,” “I’m worried about the cost,” “I don’t think it’s necessary,” and “Other people will get it so I won’t need to.”

### Statistical Analyses

We performed descriptive analysis for participant sociodemographic characteristics and COVID-19 vaccine hesitancy. Bivariate linear regression models were fitted to test associations between sociodemographic characteristics and COVID-19 KAP scores. Multivariable linear regression analyses were then conducted to further test the associations between sociodemographic characteristics and COVID-19 KAP scores while controlling for covariates found to be associated with the variables of interest in the bivariate analyses (*P*<.05). As determined a priori, variables associated with COVID-19 KAP in the bivariate analysis were retained for inclusion in the multivariable analysis. In addition, other sociodemographic variables previously found to be associated with COVID-19 KAP in the literature (ie, age, education, gender, income, gender, and marital status) were also included as covariates in the multivariable models [33-35]. All analyses were conducted in SPSS statistical software (version 9.8.0; IBM Corp).

### Ethical Approval

The study was exempted from review by the University of California, San Diego Institutional Review Board on June 22, 2020. Patients’ names or other identifying information were not collected. Participants were informed of the confidential nature of the study and were allowed to terminate the survey at any time.

## Results

### Demographics

A total of 323 participants completed the COVID-19 KAP survey. Our final data set included 265 participants after 58 individuals who did not self-identify as Latinx were excluded from analysis. Among the 265 participants, the mean age was 49.3 (IQR 38.5-59) years, and 191 (72.1%) identified as female, 139 (52.5%) were married, 109 (41.1%) indicated they had a middle school education or below, 89 (33.6%) had lost their job or were on temporary leave due to COVID-19, 92 (34.7%) had an annual income less than US $25,000, and 31 (11.7%) reported a current or previous positive test for COVID-19 (Table 1).

**Table 1:** Sociodemographic and clinical characteristics among Latinx clinic attendees in Southern California (N=265).

Variable, category			Participant
**Gender, n (%)**			
	Female	191 (72.1)	
	Male	74 (27.9)	
Age (year), mean (IQR)			49.3 (38.5-59)
**Marital status, n (%)**			
	Single/never married	84 (31.7)	
	Married	139 (52.5)	
	Divorced	10 (3.8)	
	Separated	19 (7.2)	
	Widowed	11 (4.2)	
	Refused to answer	2 (0.8)	
**Highest educational level, n (%)**			
	Middle school or below	109 (41.1)	
	High school	95 (35.8)	
	College degree	36 (13.6)	
	Graduate school	4 (1.5)	
	Refused to answer	21 (7.9)	
**Employment status, n (%)**			
	Currently employed	59 (22.2)	
	Lost job or on temporary leave due to COVID-19	89 (33.6)	
	Unemployed currently and before COVID-19	74 (27.9)	
	Retired	40 (15.1)	
	Student	2 (0.8)	
	Refused to answer	1 (0.4)	
**Annual household income (US $), n (%)**			
	<25,000	92 (34.7)	
	25,000-49,999	30 (11.3)	
	50,000-99,999	18 (6.8)	
	≥100,000	7 (2.6)	
	Refused to answer	118 (44.5)	
**Previously tested positive for COVID-19, n (%)**			
	Yes	31 (11.7)	
	No	234 (88.7)	

### COVID-19 Knowledge

Of the total 265 participants, 254 (95.8%) correctly knew that COVID-19 is spread when an infected person coughs, sneezes, or speaks; 251 (94.7%) correctly knew that COVID-19 is spread through touching contaminated surfaces and then touching the eyes or mouth; and 243 (91.7%) correctly knew to stand 6 feet or more away from another person to be safe from COVID-19 (Table S1 in Multimedia Appendix 1). However, 212 (80%) participants incorrectly believed that only older individuals (aged >65 years) are susceptible to COVID-19. Additionally, 196 (74%) participants correctly knew that individuals with pre-existing medical conditions are more susceptible to COVID-19, and 224 (84.5%) correctly identified that asymptomatic individuals with COVID-19 can still infect someone else. When asking participants for common symptoms of COVID-19, 171 (64.5%) correctly identified fever, 111 (41.9%) correctly identified cough, 109 (41.1%) correctly identified headache, 100 (37.7%) correctly identified shortness of breath, 91 (34.3%) correctly identified body aches, 46 (17.4%) correctly identified loss of taste, and 34 (13.6%) correctly identified loss of smell.

In the multivariate analysis, when adjusting for age, gender, annual household income, and education, having a college degree was significantly associated with a higher knowledge score than those who completed middle school or below (β=0.14, 95% CI 0.01-1.60; *P*=.04; Table 2).

**Table 2:** Bivariate and multivariable analysis of factors associated with COVID-19 knowledge among Latinx individuals in Southern California (N=265).

Factor		Bivariate analysis, β (95% CI)	*P* value	Multivariate analysis, aβ (95% CI)	*P* value
Age		–0.19 (–0.04 to –0.01)	.002	–0.01 (–0.03 to 0.01)	.26
**Gender**					
	Female (ref^a^)	—^b^	—	—	—
	Male	–0.04 (–0.34 to 0.72)	.30	0.06 (–0.53 to 0.56)	.95
**Marital status**					
	Married (ref)	—	—	—	—
	Single	0.01 (–0.51 to 0.55)	.93	—	—
	Divorced	0.09 (–0.24 to 1.63)	.14	—	—
	Widowed	–0.10 (–2.21 to 0.18)	.09	—	—
	Separated	0.01 (–1.18 to 1.32)	.91	—	—
**Annual household income (US $)**					
	<25,000 (ref)	—	—	—	—
	25,000-49,999	0.07 (–0.28 to 1.19)	.23	0.01 (–0.69 to 0.83)	.87
	50,000-99,999	0.14 (0.17-2.03)	.02	0.09 (–0.28 to 1.69)	.19
	≥100,000	0.13 (0.15-3.06)	.03	0.12 (–0.14 to 3.09)	.08
**Education**					
	Middle school or below (ref)	—	—	—	—
	High school	0.26 (0.23-1.23)	.005	0.11 (–0.13 to 1.01)	.25
	College degree	0.36 (0.47-1.89)	.001	0.14 (0.01-1.60)	.04
	Graduate degree	0.96 (0.46-4.24)	.02	0.12 (–0.05 to 3.92)	.07
**Employment status**					
	Lost job or on temporary leave due to COVID-19 (ref)	—	—	—	—
	Currently employed	0.08 (–0.24 to 1.03)	.22	—	.24
	Unemployed currently and before COVID-19	–0.01 (–0.63 to 0.55)	.89	—	.57
	Retired	–0.18 (–1.68 to –0.24)	.009	—	.53
	Student	–0.06 (–4.13 to 1.27)	.30	—	.19
**Previously tested positive for COVID-19**					
	No (ref)	—	—	—	—
	Yes	0.29 (–0.56 to 0.90)	.69	—	—

^a^Ref: reference.

^b^Not available (item was not accessed).

### COVID-19 Attitudes

Among the 265 total participant, 104 (39.2%) strongly disagreed or disagreed that the government would help stop the spread of the virus; 198 (74.7%) strongly agreed or agreed that they felt nervous about how the COVID-19 pandemic would impact their future; 120 (45.3%) strongly agreed or agreed that they had felt angry or frustrated because of the COVID-19 pandemic; and 147 (55.5%) strongly agreed or agreed that they had felt scared to leave their home because of COVID-19. However, a majority 72.5% (n=192) of the participants strongly agreed or agreed that they had felt hopeful about the future (Table S2 in Multimedia Appendix 1).

In the multivariate analysis, when adjusting for marital status and education, male gender had a positive association with COVID-19 attitude scores compared to female gender (β=1.61, 95% CI 0.50-2.72; *P*=.005; Table 3).

**Table 3:** Bivariate and multivariable analysis of factors associated with COVID-19 attitudes among Latinx individuals in Southern California (N=265).

Factor		Bivariate analysis, β (95% CI)	*P* value	Multivariate analysis, aβ (95% CI)	*P* value
Age		0.06 (–0.02 to 0.07)	.37	0.00 (–0.04 to 0.04)	.96
**Gender**					
	Female (ref^a^)	—^b^	—	—	—
	Male	1.65 (0.58-2.71)	.003	1.61 (0.50-2.72)	.005
**Marital status**					
	Married (ref)	—	—	—	—
	Single	–0.15 (–2.33 to –0.18)	.04	–0.13 (–2.31 to 0.08)	.07
	Divorced	0.56 (–2.74 to 1.04)	.14	–0.03 (–2.45 to 1.44)	.61
	Widowed	0.07 (–4.35 to 0.71)	.09	–0.07 (–3.99 to 1.09)	.26
	Separated	–0.09 (–3.95 to 1.11)	.91	–0.07 (–4.00 to 1.06)	.25
**Annual household income (US $)**					
	<25,000 (ref)	—	—	—	—
	25,000-49,999	–0.01 (–1.68 to 1.38)	.85	—	—
	50,000-99,999	0.02 (–1.68 to 2.17)	.81	—	—
	≥100,000	–0.01 (–3.09 to 2.93)	.96	—	—
**Education**					
	Middle school or below (ref)	—	—	—	—
	High school	–0.08 (–1.74 to 0.38)	.21	–0.06 (–1.65 to 0.73)	.45
	College degree	–0.01 (–1.55 to 1.42)	.93	0.00 (–1.64 to 1.68)	.98
	Graduate degree	0.01 (–3.85 to 4.07)	.96	–0.02 (–4.51 to 3.47)	.80
**Employment status**					
	Lost job or on temporary leave due to COVID-19 (ref)	—	—	—	—
	Currently employed	–0.09 (–1.99 to 0.46)	.96	—	—
	Unemployed currently and before COVID-19	–0.01 (–1.34 to 1.28)	.22	—	—
	Retired	0.12 (–0.21 to 2.74)	.09	—	—
	Student	0.48 (–7.73 to 3.55)	.44	—	—
**Previously tested positive for COVID-19**					
	No (ref)	—	—	—	—
	Yes	–0.05 (–2.06 to 0.88)	.43	—	—

^a^Ref: reference.

^b^Not available (item was not accessed).

### COVID-19 Preventative Practices

Among the 265 participants, when asked about preventative practices to stay safe from COVID-19 transmission, 161 (60.8%) indicated that they washed their hands most of the time when entering their homes, whereas 92 (34.7%) indicated that they never engage in this practice. Interestingly, 248 (93.6%) indicated that they always wear a mask outside of their home, whereas none indicated that they never wear a mask outside their home (Table S3 in Multimedia Appendix 1).

In the multivariable regression analysis, controlling for marital status, annual household income, and education, male gender was negatively associated with the COVID-19 practices score compared to female gender (β=–0.16, 95% CI –0.56 to –0.06; *P*=.03; Table 4).

**Table 4:** Bivariate and multivariable analysis of factors associated with COVID-19 attitudes among Latinx individuals in Southern California (N=265).

Factor		Bivariate analysis, β (95% CI)	*P* value	Multivariate analysis, aβ (95% CI)	*P* value
Age		0.04 (–0.01 to 0.01)	.54	—	—
**Gender**					
	Female (ref^a^)	—^b^	—	—	—
	Male	–0.65 (–0.56 to –0.08)	.008	–0.16 (–0.56 to –0.06)	.03
**Marital status**					
	Married (ref)	—	—	—	—
	Single	0.05 (–0.16 to 0.33)	.49	0.05 (–0.17 to 0.34)	.50
	Divorced	0.07 (–0.18 to 0.67)	.26	0.05 (–0.28 to 0.60)	.47
	Widowed	0.05 (–0.34 to 0.75)	.46	0.04 (–0.38 to 0.71)	.56
	Separated	0.02 (–0.47 to 0.66)	.74	0.03 (–0.46 to 0.71)	.67
**Annual household income (US $)**					
	<25,000 (ref)	—	—	—	—
	25,000-49,999	0.05 (–0.20 to 0.48)	.43	0.04 (–0.24 to 0.46)	.54
	50,000-99,999	–0.08 (–0.69 to 0.16)	.22	–0.04 (–0.60 to 0.33)	.57
	≥100,000	–0.01 (–0.72 to 0.62)	.88	–0.02 (–0.80 to 0.60)	.77
**Education**					
	Middle school or below (ref)	—	—	—	—
	High school	–0.01 (0.25-0.22)	.88	–0.01 (–1.65 to 0.73)	.89
	College degree	–0.11 (–0.36 to 0.30)	.87	–0.00 (–1.64 to 1.68)	.90
	Graduate degree	–0.00 (–0.90 to 0.86)	.96	0.01 (–4.51 to 3.47)	.84
**Employment status**					
	Lost job or on temporary leave due to COVID-19 (ref)	—	—	—	—
	Currently employed	0.07 (–0.15 to 0.44)	.32	—	—
	Unemployed currently and before COVID-19	0.05 (–0.18 to 0.36)	.52	—	—
	Retired	0.12 (–0.04 to 0.62)	.09	—	—
	Student	–0.07 (–1.40 to 1.08)	.80	—	—
**Previously tested positive for COVID-19**					
	No (ref)	—	—	—	—
	Yes	0.05 (–0.19 to 0.46)	.43	—	—

^a^Ref: reference.

^b^Not available (item was not accessed).

### COVID-19 Vaccine Hesitancy

We asked a subset of 203 participants about their views on a potential COVID-19 vaccine before the COVID-19 vaccine was made available. Within this subset (N=203), 111 (54.7%) indicated that they would take a vaccine if it were available, but 54 (26.6%) indicated that they would not take a vaccine, and 38 (18.7%) indicated that they were unsure (ie, answered “maybe”). Of those who indicated they would not take a vaccine (n=54), 23 (43%) indicated that they were concerned with possible side effects, 18 (33%) indicated that they didn’t think it was necessary, 8 (15%) indicated that they didn’t think it will work, and 1 (2%) indicated that they are worried about the cost and financial burden. Of those who indicated they were unsure (n=38), 27 (71%) indicated that they are concerned about possible side effects, 3 (8%) indicated that they didn’t think it will work, 2 (5%) indicated that they didn’t think it’s necessary, and none indicated that they are worried about cost and financial burden (Table 5).

**Table 5:** COVID-19 vaccine hesitancy before vaccine availability (N=203).

View on vaccine, reasons for hesitancy		Participants, n (%)
Willing to take the vaccine (n=111)		111 (100)
**Unsure about taking the vaccine (n=38)**		
	Don’t think it’s necessary	2 (5)
	Don’t think it will work	3 (8)
	Concerned about the possible side effects	27 (71)
	Worried about the cost and financial burden	0 (0)
	Refused to answer	6 (16)
**Not willing to take the vaccine (n=54)**		
	Don’t think it’s necessary	18 (33)
	Don’t think it will work	8 (15)
	Concerned about the possible side effects	23 (43)
	Worried about the cost and financial burden	1 (2)
	Refused to answer	4 (7)

## Discussion

### Principal Findings

Since the start of the COVID-19 pandemic in Southern California, the Latinx population has been disproportionately affected by disease incidence, morbidity, and mortality. Our data demonstrate that this population had good knowledge of COVID-19 transmission and spread; moderate knowledge of COVID-19 symptoms; overall negative attitudes including high pessimism in government public health efforts and high amounts fear, anxiety, and frustration due to COVID-19 pandemic; but high optimism about the future. Our data also show moderate participation in preventative practices and moderate vaccine hesitancy within a Southern California Latinx community during the first wave of the pandemic before the vaccine was available. In addition, our data show a significant association between college education and a higher COVID-19 knowledge score; male gender and a more optimistic attitude; and male gender and a lower practices score. We believe that observational data from this period are critical to identifying how a pandemic can initially affect vulnerable populations’ understanding of disease, since knowledge helps form attitudes and practices that drive behavior change [36].

### Comparison to Prior Works

Participants were particularly aware of the common physical symptoms of COVID-19 illness, such as cough, headache, and fever. Relatively few participants, however, identified loss of taste and smell as common COVID-19 symptoms. The lack of knowledge around these very specific symptoms may have been due to the unusual nature of the symptoms and the public health messaging at the time that was emphasizing “coughing” as an identifiable symptom to be aware of [37,38]. When participants were asked about their knowledge regarding COVID-19 transmission and susceptibility, most knew that transmission could happen when an infected person coughs, sneezes, or speaks and that the disease could be spread by touching contaminated surfaces and then touching the eyes or mouth. Most also knew to stand 6 feet or more away from others to prevent disease transmission. However, our data show that over half of our participants felt that only older people were susceptible to COVID-19 infection.

Our findings also demonstrate that younger age was associated with lower knowledge scores, a finding supported by Alsan et al [39], which reported that people aged <55 years were less likely to know how the disease is spread and the symptoms of COVID-19. Higher education and income were associated with higher knowledge scores, which is supported by global COVID-19 studies from 2020 that showed higher education and income allowed for greater knowledge regarding the disease [34,40,41].

When participants were asked about their COVID-19 attitudes, only one-third agreed or strongly agreed that the government would stop the spread of the virus, whereas more than half disagreed or strongly disagreed. Focus groups among Latinx farmworkers in the central valley reported similar themes of mistrust in government institutions regarding COVID-19 vaccinations and testing [42]. This finding is in-line with the Latinx community’s growing mistrust in the American government’s medical and public health policies [43].

The participants also expressed feelings of fear, anxiety, and frustration. Widespread fear, anxiety, and depression related to COVID-19 has been well-documented in the United States, and recent research has shown that these feelings have been magnified in Latinx populations where the loss of employment has been widespread [44-46]. In our study, nearly one-third of the participants reported COVID-19–related “temporary leave” or lost jobs during the pandemic. This substantial change in employment status may have also contributed to the participants’ reports of feeling angry or frustrated due to COVID-19. However, despite the negative attitudes toward COVID-19, over half of the participants indicated that they were still hopeful about the future. A positive outlook aligns with research that shows that specific Latinx populations have high resilience during stressful situations and carry optimism for the future despite dissatisfaction with the present and the multiple obstacles and stressors they face [47].

Our data show that men had more COVID-19–related positive attitudes about government response and the pandemic’s effect on their lives compared to women. Our data differ from other European- and South American–based studies that have shown no significant association between gender and COVID-19–related positive attitudes in the Latinx community [48,49]. However, our results could be explained by women having reportedly higher levels of fear and stress and negative perceptions of government actions than men during the pandemic [50]. Multiple studies conducted globally and in the United States have indicated that women have been carrying high burdens of care (eg, caring for and teaching children in the absence of in-person learning environments) and emotional labor [51-53]. Consequently, this gendered division of labor could potentially have contributed to the gender differences in positive attitudes found in our study.

Additionally, when asked about COVID-19 preventative practices, almost all participants indicated that they always wore a mask when they went outside of their home. This strong knowledge base and the corresponding safety practices regarding COVID-19 may indicate that there was effective public health messaging regarding transmission and preventative practices that had penetrated the community. In addition, local laws possibly contributed to higher compliance with COVID-19 preventative practices, such as wearing masks [54]. Our data indicate that men had decreased preventative practices compared to women. This aligns with a study by Alsharawy et al [50] indicating that women engaged in more preventative practices during the pandemic and were more risk averse.

Our data captured vaccine attitudes and acceptability between July and October 2020—a period before COVID-19 vaccines were approved for essential workers in December 2020. The most common reasons given for an expressed reluctance to accept vaccination in our study were concern about possible side effects, thinking it was not necessary, and thinking it would not work. After the vaccine was released, studies documented mass vaccine hesitancy in Latinx and Black communities across the United States who had historically faced government oppression and had mistrust in the intentions of public health medical interventions [55,56]. Our findings have built upon these studies and provided further evidence of relatively high vaccine hesitancy in marginalized populations, and we suggest that in future pandemics, more focused effort should be expended on both vaccine education and interventions that are targeted toward combatting medical mistrust in marginalized communities prior to vaccine distribution.

### Limitations

Our study had several limitations. First, due to the cross-sectional nature of the study, we are unable to report whether the KAP was casually associated with the covariates examined. In addition, we had a limited set of questions due to time constraints in the clinical setting. Lastly, the reliance on self-reporting may have introduced response bias due to participants’ fear of judgement from health care providers regarding COVID-19 practices. To mitigate these challenges, we made sure that our survey administrators were not health care providers, were well-trained, and approached surveys in a nonjudgmental manner and that the participants were informed of the confidential nature of the study and allowed to terminate the survey at any time. Additionally, our vaccine acceptance questions were asked before a vaccine was approved, hence we recognize that these findings may not reflect current attitudes toward vaccines for our population. Additionally, the cross-sectional nature of our study did not allow us to track data over time but rather enabled us to capture data at one point in time. We acknowledge that the situation with COVID-19 has changed rapidly, and therefore, KAP in populations can change over time.

We believe, however, that our data capture important information related to symptom awareness, knowledge, attitudes, and practice, in addition to vaccine hesitancy during a critical time in the history of the COVID-19 pandemic; this data might provide important insights into the future implementation of critical public health interventions and policies serving Latinx populations.

### Conclusion

We found high knowledge regarding transmission and spread; moderate knowledge regarding symptoms awareness; overall negative attitudes which included high pessimism in government public health efforts and high amounts of fear, anxiety, and frustration due to the COVID-19 pandemic; and moderate participation in preventive practices. We also found moderate vaccine hesitancy in the Latinx community in Southern California during the first wave of the pandemic. Good knowledge and preventative practices in the population may be a reflection of effective public health messaging and the implementation of public health laws during the first wave of the pandemic; however, the overall fear and anxiety may have been a reflection of the negative impact that the pandemic had on vulnerable populations such as the Latinx community. We believe it would be useful to examine KAP in larger populations during this time period and potentially use the data to inform public health approaches in Latinx communities amid the potential arrival of new pandemics.

## Appendix

Multimedia Appendix 1 Additional knowledge, attitude, and practices survey results.

## Abbreviations

KAP: knowledge, attitude, and practices
WHO: World Health Organization
