# Supplementary material for: Knowledge, Attitude, Practices, and Vaccine Hesitancy Among the Latinx Community in Southern California Early in the COVID-19 Pandemic: Cross-sectional Survey
Source: JMIR Form Res. 2022 Aug 4;6(8):e38351. doi: 10.2196/38351 (PMC9359308; doi:10.2196/38351)
Supplement: Multimedia Appendix 1 [file formative_v6i8e38351_app1.docx]

**Multimedia Appendix**

**Table S1:** Knowledge of COVID-19

| **Knowledge Questions** | **Frequency (%) (N=265)** | |
| --- | --- | --- |
| **Transmission and Spread** | Correct | Incorrect |
| Can people be infected with COVID-19 when an infected person coughs, sneezes, or speaks? | 254 (95.8%) | 11  (4.2%) |
| Does COVID-19 spread through touching contaminated surfaces and then touching the eyes or mouth? | 251  (94.7%) | 14  (5.3%) |
| How far should you be from another person to be safe from COVID-19? | 243  (91.7%) | 22  (8.3%) |
| Do only older people (65+ years) get severe COVID-19 infection? | 53  (20.0%) | 212  (80.0%) |
| Are people with pre-existing medical conditions (such as diabetes, hypertension, obesity) more likely to experience serious medical problems from COVID-19? | 196  (73.9%) | 69  (26.1%) |
| Can an infected person who does not have symptoms of COVID-19 infect someone else? | 224  (84.5%) | 41  (15.5%) |
| **Symptom Awareness “What are the Common Symptoms of COVID-19?”** |  |  |
| Cough | 111  (41.8%) | 154  (58.2%) |
| Headache | 109  (41.1%) | 156  (58.9%) |
| Shortness of Breath | 100  (37.8%) | 165  (62.2%) |
| Body Aches | 91  (34.3%) | 174  (65.7%) |
| Fever | 171  (64.5%) | 94  (35.5%) |
| Loss of Taste | 46  (17.3%) | 219  (82.7%) |
| Loss of Smell | 34  (12.8%) | 231  (87.2%) |

**Table S2:** Attitudinal Statements Regarding COVID-19

| **Attitudinal Statements** | **Frequency (%) (N=265)** | | | | |
| --- | --- | --- | --- | --- | --- |
|  | Strongly Disagree | Disagree | Neutral | Agree | Strongly Agree |
| I think that the government will stop the spread of the virus. | 53  (20.0%) | 51  (19.2%) | 55  (20.6%) | 47  (17.6%) | 60  (22.6%) |
| I feel nervous about how COVID-19 will impact my future. | 25  (9.4%) | 16  (6.0%) | 26  (9.9%) | 73  (27.6%) | 125  (47.1%) |
| I am feeling angry or frustrated because of COVID-19. | 81  (30.6%) | 29  (10.9%) | 35  (13.2%) | 64  (24.1%) | 56  (21.2%) |
| I feel scared to leave my home because of COVID-19. | 46  (17.6%) | 45  (17.2%) | 27  (10.2%) | 66  (24.9%) | 81  (30.1%) |
| I feel hopeful about the future. | 18  (6.8%) | 17  (6.4%) | 38  (14.3%) | 77  (29.3%) | 115  (43.2%) |

**Table S3:** Practices Related to COVID-19

| **Practice Questions** | **Frequency (%) (N=265)** | | | | |
| --- | --- | --- | --- | --- | --- |
|  | Never | Rarely | Sometimes | Most of the time | Always |
| Do you wash your hands when you come into your house from outside? | 92 (34.7%) | 2  (0.7%) | 0  (0.0%) | 161  (60.7%) | 10  (3.9%) |
| Do you wear a mask when you go outside your home? | 0  (0%) | 2  (0.7%) | 2  (0.7%) | 13  (4.9%) | 248  (93.7%) |
